# Short-Term Outcomes of Transabdominal Preperitoneal Ventral Hernia Repair With Rectus Aponeuroplasty (TAPPRA) for the Management of Incisional Hernias

**DOI:** 10.3389/jaws.2024.13195

**Published:** 2024-09-11

**Authors:** Maggie E. Bosley, Zev Felix, Gustavo Salgado-Garza, Shan Lansing, Vahagn C. Nikolian

**Affiliations:** Department of Surgery, Oregon Health & Science University, Portland, OR, United States

**Keywords:** ventral hernia repair, extraperitoneal mesh placement, preperitoneal mesh repair, robotic abdominal wall surgery, rectus aponeuroplasty

## Abstract

**Introduction:**

Options for minimally invasive ventral hernia repair continue to evolve as a function of our understanding of the abdominal wall and the development of new techniques. We describe a robotic transabdominal pre-peritoneal repair with concurrent rectus aponeuroplasty (TAPPRA) for incisional and recurrent ventral hernias.

**Methods:**

All patients in this retrospective cohort study underwent TAPPRA repair between October 2023 and March 2024. This study aimed to determine intraoperative feasibility of the technique and to assess immediate postoperative outcomes.

**Results:**

Twelve patients underwent TAPPRA repair for incisional and/or recurrent ventral hernias at an academic hernia center. The median case duration was 135 min with no significant intraoperative complications noted. Average defect size for the hernias measures 6.5 × 8.5 cm. Polypropylene mesh was used to reinforce all defects, with the average dimensions being 19.7 × 21.5 cm. 83% of patients were discharged within 24 h of their procedure. No significant postoperative complications were noted.

**Conclusion:**

We describe the first use of a novel ventral hernia repair technique, TAPPRA, and demonstrate that it is safe, feasible, and associated with appropriate short-term outcomes for repair of moderate sized incisional hernias.

## Introduction

Ventral hernia repair is one of the most commonly performed procedures in general surgery [1]. The approach to repair has evolved dramatically in the modern era. Hernia repair strategies have pushed towards the utilization of broad, extraperitoneal positioned mesh with a premium placed on defect closure [2, 3]. The advent of modern techniques such as transversus abdominis release (TAR), have provided safe and reproducible approaches for achieving broad mesh coverage for complex reconstruction, while simultaneously reducing wound morbidity traditionally present for advanced procedures such as anterior component separation [4–6]. Further enhancing surgeons’ ability to deliver complex repair options while minimizing morbidity has been the revolutionary robotic approaches to abdominal wall reconstruction, which have been allowed surgeons to attain comparable long term outcomes and reduce morbidity further [4, 5].

Minimally invasive abdominal wall reconstruction is in its early phase of adoption. Spurred by procedures like intraperitoneal onlay mesh repair (IPOM), transabdominal preperitoneal repair (TAPP), and enhanced totally extraperitoneal techniques (eTEP), general surgeons have expanded their armamentarium to address ventral hernia defects [7]. Expert opinion on the best technique for a given defect is diverse and dependent on a variety of factors. However, one common and emerging theme among abdominal wall specialists is the realization that hernias may be considered a chronic disease process, and that abdominal wall planes should be preserved to allow for future operations to be performed. Beyond preservation of hernia repair options for the future, many experts have noted that over utilization of advanced techniques such as TAR, can be associated with major complications [8].

We sought to develop a technique to allow for broad mesh coverage, decrease tension on fascial closure while minimizing the potential for injuries to the neurovascular elements of the abdominal wall. As such, an extended transabdominal preperitoneal dissection with concurrent rectus aponeuroplasty (TAPPRA) was developed. We share our initial experience and the development of the TAPPRA technique. Further, we describe early perioperative outcomes for patients who have had this operation. Our study aims to assess the safety, efficacy, and results of TAPPRA technique for moderate sized ventral hernia defects.

## Methods

### Hernia and Abdominal Wall Center

All procedures were performed at an academic hernia and abdominal wall center in the Pacific Northwest of the United States. The operation was developed and performed by the same surgeon (VCN). The operations were performed using the Intuitive DaVinci Xi robotic platform (Intuitive Surgical, Sunnyvale, CA United States).

### Data Collection

A prospective maintained database for all patient undergoing hernia repair has been established by our center. Patient demographics were collected including body mass index (BMI), prior hernia repair attempts, and common comorbidities. Intraoperative variable including estimated blood loss, case duration, mesh type and size, fixation strategy, and suture types were collected. Short-term outcomes related to length of stay, postoperative complications, and procedural interventions were assessed. Given the fact that this was a feasibility study, long term data related to our surveillance was limited and has not been included in this initial review.

### Surgical Technique

TAPPRA technique is a derivative of two commonly performed and well described techniques in ventral hernia repair. It combines preperitoneal dissection for extraperitoneal mesh positioning with intracorporeal rectus aponeuroplasty [9, 10]. These previous techniques have typically been used for smaller defects, but we have aimed to apply it towards moderate hernia defects ranging in size from 4–10 cm in transverse dimension.

The operation is initiated with abdominal access and placement of lateral robotic trocars. A peritoneal incision is made 5–7 cm from the ipsilateral hernia defect margin. A preperitoneal dissection is conducted, taking advantage of the falciform and periumbilical fat. The preperitoneal dissection is carried to the contralateral abdominal wall, often extending to the contralateral retroperitoneum. Upon completing the dissection, defect closure is performed. To facilitate closure and minimize tension on the fascial closure, a posterior rectus sheath aponeuroplasty is performed. The posterior sheath is identified and incised roughly 1 cm from the linea alba. No dissection is performed in the retrorectus space, to preserve this area and minimize injuries to the neurovascular elements present in the retrorectus space. Fascial closure is completed typically with a #1 permanent barbed suture. Following closure, the decision is made for either double docking the robotic trocars on the contralateral abdomen to perform an extended preperitoneal dissection and accommodate a large mesh, or to maintain the original docking position and perform a less extensive retrograde pre-peritoneal dissection on the ipsilateral side. For larger defects (generally, greater than 7 cm in width), our team favors using broad mesh coverage and will readily double-dock the robotic platform and perform preperitoneal dissection. Depending on the decision for single unilateral docking vs. double docking, mesh and peritoneum are managed in the following ways:

1) Single site docking: Macroporous polypropylene mesh is placed fixated to the anterior abdominal wall with interrupted stitches using 3-0 fast-absorbing suture. The peritoneal flap is closed using a 3-0 slow absorbing V-loc suture. Fenestrations in the flap are identified and closed.
2) Double site docking: the visceral sac is reconstructed and all fenestrations closed. Once completed, mesh is placed and opened above the peritoneal flap and the space is de-sufflated.
  Representative videos of these techniques from VCN may be viewed in the following links:
1) Single dock:^1^
2) Single dock with case set up:^2^
3) Double dock for moderate defect:^3^
4) Double-dock for larger defect:^4^

**FIGURE 1:**
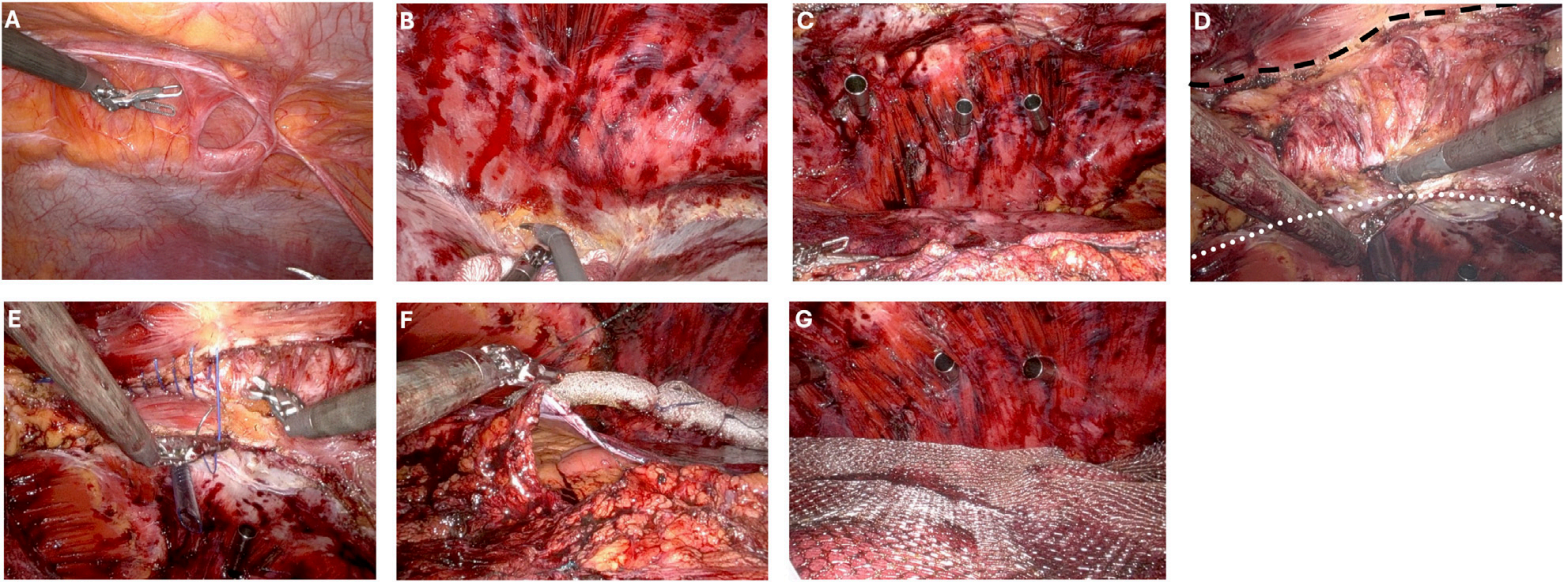
Critical steps of double-dock transabdominal preperitoneal ventral hernia repair with rectus aponeuroplasty **(A)** Ventral defect prior to dissection. **(B)** Preperitoneal dissection to contralateral pretransversalis space. **(C)** Placement of contralateral trocars in preperitoneal/pretransversalis space. **(D)** Rectus aponeuroplasty with dotted line defining the line of transection lateral to the linea alba. Dashed line along the ipsilateral rectus margin with completed rectus aponeuroplasty and exposed rectus muscle. **(E)** Anterior fascial defect closure. **(F)** Visceral sac closure. **(G)** Mesh placement.

Key steps in the operation are depicted in Figure 1.

### Postoperative Care

At the completion of the case, patients were extubated after reversal of paralysis using Sugammadex. Patients are provided an abdominal binder and encouraged to wear compression garments in the initial postoperative period. Multimodal pain control strategies are utilized, with a preference for non-narcotic analgesics. Same-day discharge is the anticipated postoperative plan, but all procedures are performed in venues that have the possibility of admitting patients for post-operative observation. Initial follow up occurs within a few weeks of the operative date. All patients are enrolled in our hernia surveillance program to assess for long-term outcomes [11].

### Institutional Review Board

This research secured approval from our institutional review board at Oregon Health & Science University, Portland, OR. Given the retrospective nature and minimal risk classification of this study, patient consent was determined to be unnecessary and implied.

## Results

### Preoperative Patient Factors

TAPPRA ventral hernia repair technique was used to address ventral hernias in 12 patients during the observation period. During that time period, 202 total ventral hernia repair operations were performed, resulting in a use rate of 5.9%. The average age of patients undergoing TAPPRA was 61 years (range 44–79 years), with an average BMI of 31.5 kg/m2. Indications for surgery included incisional hernias (10 patients, 83%) and recurrent hernias (1 patient, 8.3%). Patient modifiable comorbidities included BMI >30 kg/m2 (n = 7, 58%). A summary of patient factors is provided in Table 1.

**TABLE 1:** Preoperative patient specific factors.

Factors	Total patients N = 12 (n%)
Age, years; mean (range)	60.75 years (44–79 years)
Male gender	7 (58%)
Primary Insurance	
Medicare	6 (50%)
Private	6 (50%)
Incisional hernia	10 (83%)
Recurrent hernia	1 (8.3%)
Modifiable Risk Factors	
BMI; mean (range)	31.5 (23–42)
BMI >25	9 (75%)
BMI >30	7 (58%)
Diabetes, HbA1c > 7%	0 (0%)
HTN	6 (50%)
Anti-platelet medications	3 (25%)
Anti-coagulation medications	13 (6%)

### Intraoperative and Hernia Specific Variables

Intraoperative factors were reviewed. The majority of patients presented with defects centered around the umbilicus (M3, per European Hernia Society nomenclature) [12]. The average defect size measured 6.5 cm by 8.6 cm, with the large defect width being 10 cm. Operations were performed via unilateral port placement (docking) in 75% of cases. The remaining operations to address larger defects (generally, greater than 7 cm in width) were managed with a double docking strategy. Mesh preference in these cases was for macroporous polypropylene mid-weight mesh (10 patients, 83%). The average mesh size was 19.7 × 21.5 cm (range 12–30 cm in transverse, 15–30 cm in cranial-caudal dimension). A summary of the intraoperative factors is provided in Table 2.

**TABLE 2:** Intraoperative patient specific factors.

Factors	Total patients N = 12 (n%)
European Hernia Classification Hernia Location^a^	
M1	1 (8.3%)
M2	5 (42%)
M3	12 (100%)
M4	5 (42%)
Defect width, average (range)	6.5 cm [4–10]
Defect length, average (range)	8.6 cm [4–16]
Operative time (min), mean (range)	135 min (60–252)
Operative Approach	
Single dock	9 (75%)
Double dock	3 (25%)
Mesh use	
Macroporous polypropylene (mid-weight)	10 (83%)
Microporous polypropylene (heavy-weight)	2 (17%)
Mesh width, average (range)	19.7 cm (12–30)
Mesh length, average (range)	21.5 cm (15–30)
Mesh fixation strategy	
Suture to anterior wall	9 (75%)
Fibrin sealant to visceral sac	1 (8.3%)
None	2 (16.7%)
Drain placement	
Adjacent to mesh in preperitoneal plane	3 (25%)
Subcutaneous drain	1 (8.3%)

^a^ Some patient with hernias that spanned multiple regions of abdominal wall.

### Postoperative Outcomes

Given the novel nature of this operative technique, we have limited data on long term outcomes. The majority of patients (n = 8, 66%) were discharged on the same day of their procedure. Only 2 patients (17%) required hospitalization beyond one night. Surgical site occurrences and/or procedural interventions were not required in any patient and narcotic utilization was less than 10 tablets/patient for all but 1 patient (91.7% narcotic free). The median length of surveillance was 7 months, with a range of 3–9 months for all participants. A summary of postoperative outcomes is provided in Table 3.

**TABLE 3:** Postoperative patient outcomes.

Outcomes	Total patients N = 12 (n%)
Length of Stay	
Same day discharge	8 (66%)
Overnight observation	2 (17%)
2-3 nights	2 (17%)
Surgical site occurrences (SSO)	0 (0%)
Procedural interventions	0 (0%)
Narcotic utilization^a^	
No narcotic utilization	8 (66.7%)
1–10 tablets	3 (25%)
>15 tablets	1 (8.3%)
Short-term Follow-up (y/n) at 3 months	12/12 (100%)
Clinical recurrence	0 (0%)
Follow up duration, median (range)	7 (3–9) months

^a^ all patients discharged with oxycodone 5 mg tablets.

## Discussion

The optimal surgical approach for moderate sized ventral hernias is complex and dependents on multiple patient, surgeon, and institutional factors. We report our initial experience of a novel technique for robotic ventral hernia repair, TAPPRA, and demonstrate that short-term outcomes are appropriate with expected findings related to intraoperative reproducibility and immediate postoperative outcomes. We are encouraged by these findings which allow for yet another technique to minimally invasive hernia repair with ventral defect closure and broad mesh reinforcement.

Contemporary ventral hernia repair has evolved dramatically in the last two decades. Many of the principles of hernia repair have been derived from retromuscular techniques proposed by Rives and Stoppa—namely prosthetic reinforcement of the visceral sac [13]. Over the decades, this approach has allowed for multiple iterative changes that have led to the modern day hernia practice [14]. Traditional open ventral hernia repair with myofascial advancement techniques have been associated with good outcomes in select populations. However, these techniques carried high rates of wound complications and perioperative morbidity. As such, surgeons explored minimally invasive techniques such as IPOM, with a goal of minimizing wound complications [7]. IPOM, though effective in the short term, had many weaknesses as defect closure was often not achieved. Further, the ability for surgeons to use IPOM to address hernias in atypical locations was poor, as circumferential penetrating fixation was required for success [9]. As a function of robotic technology, and expansion of our working understanding of the abdominal wall, newer techniques/approaches such as IPOM+, LIRA, rTAPP, and eTEP have since been introduced which allow for ventral hernia repair that results in more durable outcomes than IPOM [7, 10]. Our proposed procedure takes elements of these operations, specifically LIRA and rTAPP, to allow for surgeons to address moderate defects with an extraperitoneal mesh.

TAPP ventral hernia repair is now a common procedure among surgeons. For smaller defects, it serves a valuable role, allowing for defect closure, extraperitoneal mesh, and minimizes the need for penetrating fixation of mesh [9]. However, for larger defects, the TAPP approach can be associated with excessive tension during defect closure. For many surgeons, rather than attempting a preperitoneal repair for moderate defects, a retromuscular repair is conducted, which results in medialization of the rectus abdominis and anterior fascia through a variety of maneuvers [15]. The process of myofascial advancement in these techniques has been attributed to many factors, including incision of the posterior rectus sheath, dissection of the retrorectus space, incision of the posterior lamella of the internal abdominal oblique aponeurosis, and for advanced procedures, transection of the transversus abdominis muscle and subsequent dissection in the pretransversalis space [16, 17]. Unfortunately, complications related to retromuscular repair are significant and can burn many bridges to future repair [18]. Our described technique predominantly performs a dissection the preperitoneal plane and has a limited retromuscular component. By performing rectus aponeuroplasty, we are able to off-load tension, allowing for closure of larger defects.

Extended preperitoneal dissections have been described for many decades, but have been considered to be challenging and difficult to reproduce [19]. Outcomes at high volume centers have demonstrated iterative improvement in this technique. As general surgeons have become more comfortable with retromuscular dissection, this complex technique now appears to be more attainable. We find that TAPPRA is a reproducible option for hernia repair that does not obviate any other options for reconstruction. The operation may proceed with unilateral docking or a double docking technique. The double docking strategy is typically reserved for wider defects that will require broad mesh coverage. Rather than a traditional TAR procedure, this extended preperitoneal dissection allows for extension of the dissection beyond the semilunar line without disrupting the muscular and aponeurotic elements of the abdominal wall. Similar strategies have been implemented by the Madrid group, but often require a retrorectus dissection which may result in increased potential for neurovascular trauma to the abdominal wall [20].

This study has several notable limitations. First, it is a retrospective review of select cases performed at a high-volume hernia center. The surgeon included in this study has completed a formal fellowship in abdominal wall reconstruction and has overcome their initial learning curve in robotic ventral hernia repair. Second, this study exclusively evaluates feasibility of performing these repairs with limited information on long-term outcomes. Though those findings will be paramount, this technique is a derivative of commonly performed operations that are well described in the hernia literature and have demonstrated acceptable long-term results. We have been encouraged by the low rate of short-term complications and look forward to long-term follow up with these patients. Third, the operations are performed using robotic surgical platform. Though these techniques can be performed with traditional laparoscopic instruments, robotic assisted surgery facilitates management of complex peritoneal flaps and may make broad adoption challenging. More robust evaluation of this technique is necessary. We are currently collaborating with other hernia centers to evaluate the utility of this technique and compare it to other MIS techniques for ventral hernia repair.

In summary, we report our first experience of performing robotic TAPPRA technique for the management of moderate sized ventral hernias. This technique appears to be associated with a low complication profile and can be applied to many patients. Future studies evaluating the optimal patient selection strategy and long-term outcomes will be important in determining if this technique can be more broadly applied.

## Data Availability

The raw data supporting the conclusions of this article will be made available by the authors, without undue reservation.

## Footnotes

1
https://youtu.be/hax1A6TxdnY?si=eJTuB_QJ5Uv201Tm

2
https://youtu.be/s1ly18jbt6Q?si=PqaGka45iFIfpI9b

3
https://youtu.be/XG7iIPkdXvA?si=vyzydbxp3YTWFtWl

4
https://youtu.be/sZ_Wt_okoL4?si=GeSwztpabHaet7Q-
